# Erythropoietic Potential of CD34+ Hematopoietic Stem Cells from Human Cord Blood and G-CSF-Mobilized Peripheral Blood

**DOI:** 10.1155/2014/435215

**Published:** 2014-05-05

**Authors:** Honglian Jin, Han-Soo Kim, Sinyoung Kim, Hyun Ok Kim

**Affiliations:** ^1^Division of Transfusion Medicine and Cell Therapy, Department of Laboratory Medicine, Yonsei University College of Medicine, 50 Yonsei-ro, Seodaemun-gu, Seoul 120-752, Republic of Korea; ^2^Innovative Cell and Gene Therapy Center, International St. Mary's Hospital, 25 Simgok-ro, 100 beon-gil, Seo-gu, Incheon 404-834, Republic of Korea

## Abstract

Red blood cell (RBC) supply for transfusion has been severely constrained by the limited availability of donor blood and the emergence of infection and contamination issues. Alternatively, hematopoietic stem cells (HSCs) from human organs have been increasingly considered as safe and effective blood source. Several methods have been studied to obtain mature RBCs from CD34+ hematopoietic stem cells via *in vitro* culture. Among them, human cord blood (CB) and granulocyte colony-stimulating factor-mobilized adult peripheral blood (mPB) are common adult stem cells used for allogeneic transplantation. Our present study focuses on comparing CB- and mPB-derived stem cells in differentiation from CD34+ cells into mature RBCs. By using CD34+ cells from cord blood and G-CSF mobilized peripheral blood, we showed *in vitro* RBC generation of artificial red blood cells. Our results demonstrate that CB- and mPB-derived CD34+ hematopoietic stem cells have similar characteristics when cultured under the same conditions, but differ considerably with respect to expression levels of various genes and hemoglobin development. This study is the first to compare the characteristics of CB- and mPB-derived erythrocytes. The results support the idea that CB and mPB, despite some similarities, possess different erythropoietic potentials in *in vitro* culture systems.

## 1. Introduction

Red blood cell transfusion is a well-established and essential therapy for patients with severe anemia. However, the worldwide supply of allogeneic blood faces a serious shortage, and there are many patients around the world whose survival depends on blood transfusion. Around 92 million blood donations are collected annually from all types of blood donors (voluntary unpaid, family/replacement, and paid), but in the report of 39 counties of 159 countries on their collections, donated blood is still not routinely tested for transfusion-transmissible infections (TTIs) including HIV, hepatitis B, hepatitis C, and syphilis [1]. Nevertheless, blood transfusion saves lives, but the transfusion of unsafe blood puts lives at risk because HIV or hepatitis infections can be transmitted to patients through transfusion. However, the financial consequence of discarding unsafe blood creates yet another burden in developing countries.

Research performed on stem cells, specifically hematopoietic stem cells (HSCs), holds promise for the production of mature red blood cells in large quantities through differentiation induction. The classic source of HSCs has been the bone marrow, but bone marrow procurement of cells is an invasive process with risks. The artificial RBCs from stem cells* in vitro* culture can be generated from sources such as embryonic stem cells (ESCs) [2], induced pluripotent stem cells (iPSs) [3], cord blood (CB) [4–6], and peripheral blood (PB) [7]. Of these, ESCs and iPSCs are the least promising due to the low generation efficiency and long-term* in vitro* culture cost hindrances. Currently, granulocyte colony-stimulating factor- (G-CSF-) mobilized peripheral blood (mPB) and CB are therefore widely researched as a potential alternate source for stem cell procurement. However, this has not been a widespread standard of therapy, and the characteristics of mature red blood cells derived from HSCs after mass production are not yet well known. Our study focuses on comparing CB- and mPB-derived stem cells with respect to their characteristics and function after differentiation.

## 2. Materials and Methods

### 2.1. CD34+ HSC Isolation, Culture, and Erythropoietic Differentiation

CB samples from normal full-term deliveries (*n* = 7) were collected in a bag (Green Cross Corp., Yong-in, Korea) containing 24.5 mL of citrate phosphate dextrose A (CPDA-1). Five milliliters of G-CSF-mPB was obtained (*n* = 7) with the written informed consent of normal voluntary allogeneic HSC donors. This study was approved by Severance Hospital IRB (IRB number 4-2011-0081). The CD34+ cells from both sources were isolated using a MACS isolation kit (density, 1.077; Pharmacia Biotech, Uppsala, Sweden) using an antibody against CD34 according to the manufacturer's instructions. And the sorted CD34+ cells were cultured at a density of 1 × 10^5^ cells/mL in a stroma-free condition for 17–21 days as described previously [8, 9]. Briefly, from day 0 to 7, sorted CD34+ cells were continually cultured in serum-free conditioned erythrocyte culture medium with 100 ng/mL SCF (Peprotech, Rehovot, Israel), 10 ng/mL IL-3 (Peprotech), and 6 IU/mL recombinant EPO (Recormon Epoetin beta, Roche) with a half-volume medium change twice a week. Serum-free culture medium consisted of StemPro-34 SFM Complete Medium (Gibco, Grad Island, NY) supplemented with 1% bovine serum albumin (Sigma), 150 *μ*g/mL iron-saturated human transferrin (Sigma), 50 *μ*g/mL insulin (Sigma), 90 ng/mL ferrous nitrate (Sigma), 2 mMol/L L-glutamine (Sigma), 1.6 × 10^−4^ mol/L monothioglycerol (Sigma), 30.8 *μ*M/L vitamin C (Sigma), 2 *μ*g/mL cholesterol (Sigma), and 1% penicillin-streptomycin solution (Gibco). In the second 7-day period of culture, the medium was replaced with serum-free conditioned medium with 3 IU/mL of recombinant EPO, 50 ng/mL of SCF, and 10 ng/mL of IL-3 for expansion and differentiation. During days 15–18 of culture, only one cytokine (EPO, at 2 IU/mL) was used for erythrocyte differentiation, and poloxamer 188 (Pluronic F68 (F68), Sigma; MW 8400) was added at a concentration of 0.05%. No cytokines were added during days 19–21 of culture, and only poloxamer 188 was added during this period. At the end of each phase, cultured cells were counted using a hemocytometer. The trypan blue stain was used in all cell counts, and only viable cells are included in the fold expansion results. All cultures were maintained at 37°C in a humidified atmosphere of 5% CO_2_.

### 2.2. Assessment of Cell Morphology

Cell morphology was assessed using slides prepared by Cytospin using a cytocentrifuge (Cytospin 3, Shandon Scientific, Tokyo, Japan) at 800 rpm for 4 min followed by Wright-Giemsa staining. Pictures of the stained cells were taken with a digital camera (DP70, Olympus, Tokyo, Japan) at 400x magnification.

### 2.3. Differential Counting of Cultured Erythroblasts

Five differential countings were enumerated as proerythroblasts, early and late basophilic erythroblasts, polychromatic erythroblasts, and orthochromatic erythroblasts at 1000x magnification.

### 2.4. Flow Cytometric Analyses of Erythroid Markers

For flow cytometric analyses of cell surface antigens, a total of 1 × 10^5^ cells were stained with phycoerythrin- (PE-) or fluorescein isothiocyanate- (FITC-) conjugated mouse anti-human antibodies against CD45, CD34, CD71, and glycophorin A (GpA) for 15 min, washed, resuspended in FACS buffer, and analyzed using a Cell Lab Quanta SC (Beckman Coulter, Fullerton, CA, USA) using a 488 nm wavelength laser. Cells were analyzed using two-color flow cytometry through WinMDI 2.9. The antibody combinations used were CD45-FITC/CD34-PE and CD71-FITC/GpA-PE, using G1-FITC/G1-PE as a control. All fluorescent conjugated monoclonal antibodies used were purchased from BD Biosciences (San Jose, CA).

### 2.5. Quantitative Real-Time Polymerase Chain Reaction

To evaluate gene expression levels during erythrocyte differentiation from different sources, we harvested over 1 × 10^6^ cells from cultured erythrocytes at 7, 10, 14, and 17 days and isolated total RNA for quantitative polymerase chain reaction (PCR). Gene expression levels were quantified using the Light Cycler 480 Real-time PCR System (Roche Applied Science). Quantitative real-time polymerase chain reaction (qPCR) was performed using Light Cycler 480 SYBR Green I Master mix (Roche Applied Science) according to the manufacturer's instructions. Primers were designed [10, 11] and generated by Bioneer (Korea) (Table 1). Total RNA (800 ng) was used to generate first-strand cDNA using the Maxime RT Premix Kit (Intron Biotech). Differences between the Cp (crossing point) values of actin and target mRNAs for each sample were used to calculate ΔCp values. The ΔCp values derived from the isolated, undifferentiated CD34+ cells were used as control ΔCp values. Relative expression levels between samples and controls were determined using the formula: relative expression level = 2^−(*S*ΔCp−*C*ΔCp)^. Comparative real-time PCR with primers specific for GATA1, GATA2, EKLF, eALAS, and SCL/Tall (Table 1) was performed in triplicate. Reactions were performed at 95°C for 10 min, followed by 45 cycles of 95°C for 30 s, 60°C for 30 s, and 72°C for 30 s.

### 2.6. Functional Analysis of Hemoglobin

We used a Hemox-Analyzer (TCS, Medical Products Division, Southampton, PA) to measure the oxygen binding and dissociation abilities of the hemoglobin produced in mature erythrocytes derived from the mPB and cord blood. Hemox-Analyzer is an automatic system for recording blood oxygen equilibrium curves and related phenomena [12]. The operating principle of the Hemox-Analyzer is based on dual-wavelength spectrophotometry for the measurement of the optical properties of hemoglobin and a Clark electrode for measuring the oxygen partial pressure in millimeters of mercury. The resulting signals from both measuring systems are fed to the *X*-*Y* recorder. Both the P_50_ value and observation of the fine structure of the curve can furnish information about the delivery of oxygen to tissues. CD34+ cells derived from CB and mPB that were cultured for 17 days in three separate phases were analyzed using this system. Normal red blood cells were used as a control.

### 2.7. Capillary Zone Electrophoresis

After 17 days of culture, 1 × 10^8^ cells were collected and assessed by capillary zone electrophoresis. Capillary zone electrophoresis was performed as described previously using the Sebia Capillary system (Sebia, Norcross, GA) [13]. Differentiated erythrocytes (5 × 10^7^cells) were centrifuged at 5,000 rpm for 5 minutes. Thereafter, the culture medium was removed, and the erythrocyte pellet was vortexed for 5 s. Electrophoresis was performed in alkaline buffer (pH 9.4) provided by the manufacturer (Sebia), with separation primarily due to the pH of the solution and endosmosis. The hemoglobin was measured at a wavelength of 415 nm. Electrophoretograms were recorded with the location of specific hemoglobin in specific zones.

### 2.8. Statistical Analysis

Student's *t*-test was performed using Excel (Microsoft). *P* values less than 0.05 were considered statistically significant.

## 3. Results 

### 3.1. *In Vitro* Culture Supports the Differentiation of Erythrocytes

**Figure 1:**
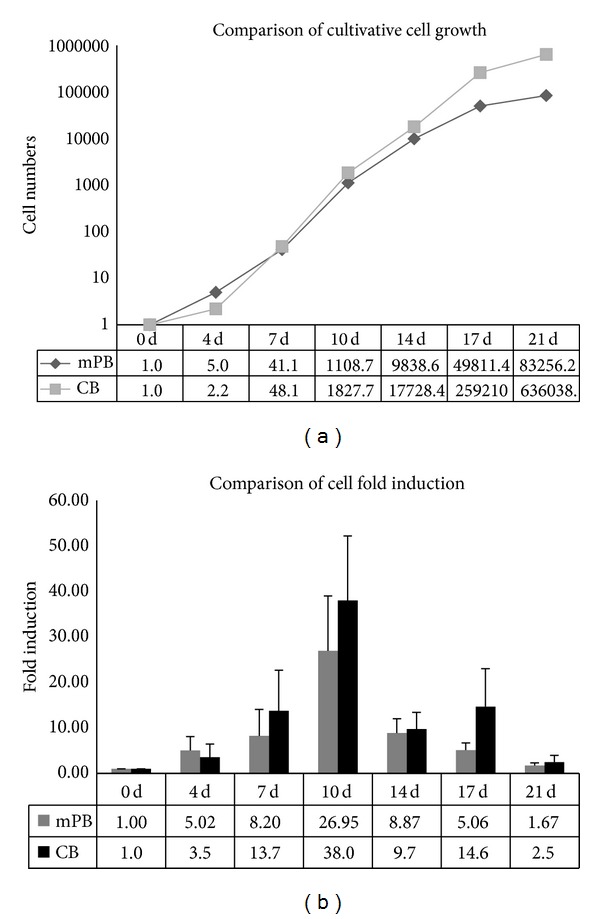
Comparison of cell growth in PB- and CB-derived CD34+ cell cultures. Stem cells were cultured for 21 days and counted at the end of each phase. (a) Erythroid cell amplification (mean ± standard deviation) of mPB- and CB-derived CD34+ cells. (b) Numbers of total cells expanded from cultures of mPB- and CB-derived CD34+ cells. CB-CD34+ cells exhibit higher amplification efficiency than mPB cells. *P< 0.001.

**Figure 2:**
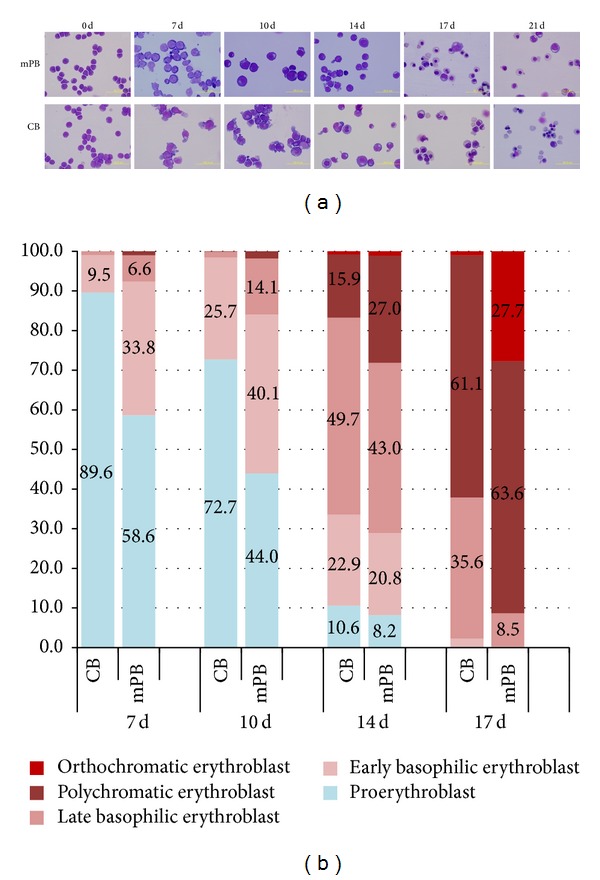
Comparison of cell morphological changes in mPB- and CB-derived CD34+ cell cultures. CD34+ cells selected from mPB and CB were cultured for 21 days* in vitro*. Morphological changes in differentiated cells from both sources were similar. No remarkably different patterns were found in the Giemsa staining photos (a). Based on the erythrocyte cell type counting at the end of each phase, the mPB-derived cells matured more rapidly than the cells derived from CB, even though cells from both sources have similar maturation patterns (b).

The number of cell divisions observed significantly increased during the second phase of the culture period. Compared to mPB-CD34+ cells, CB-CD34+ cells have greater proliferative capacity during days 10–21of culture (Figure 1(a)). This difference led to CB cultures achieving a greater total number of cells than that of mPB cultures (636,038 ± 182,817 versus 83,256 ± 8,858). Cell growth in CB cell cultures exceeded that of mPB cell cultures in the second phase and early third phase of culture (Figure 1(b)). Following erythropoietic differentiation, decreased cell size, nuclear condensation, and nuclear extrusion were confirmed by Wright-Giemsa staining. Although there were no significant differences found, our results show that, in the blood type composition count, the CB HSCs have more multipotency while mPB-CD34+ cells show earlier differentiation into mature erythrocytes (Figure 2(a)). These results demonstrate that mPB- and CB-derived CD34+ HSCs have similar growth patterns and morphological characteristics, but mPB-derived CD34+ cells show faster maturation than CB-derived CD34+ cells (Figure 2(b)).

### 3.2. Similar Immunophenotypic Patterns

**Figure 3:**
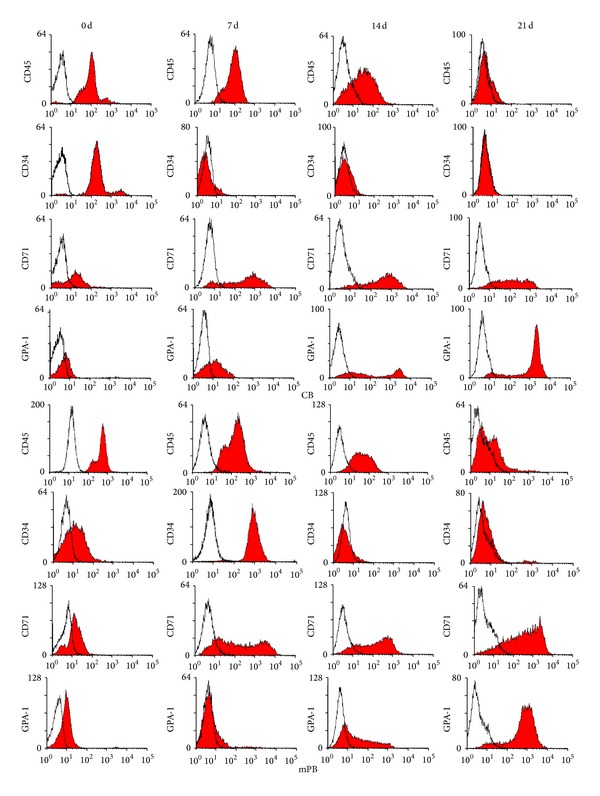
Phenotypic markers in erythrocytes differentiated from mPB- and CB-derived CD34+ cells. Cells (3 × 10^5^) from the end of each phase of culture were stained, and hematopoietic and erythropoietic markers were measured. Flow cytometry results show similar patterns in the cultured cells from both sources and no significant differences.

CD34 and CD45 marker dramatically decreased and finally disappeared from the cells during differentiation, while GPA expression increased during the 21 days of culture. Although CD71 expression increased until early in the third phase of culture, it gradually decreased following final maturation (Figure 3). From the immunophenotypic data, we found no significant differences between mPB- and CB-derived CD34+ cells during erythroid cell maturation.

### 3.3. Different mRNA Expression Levels

**Figure 4:**
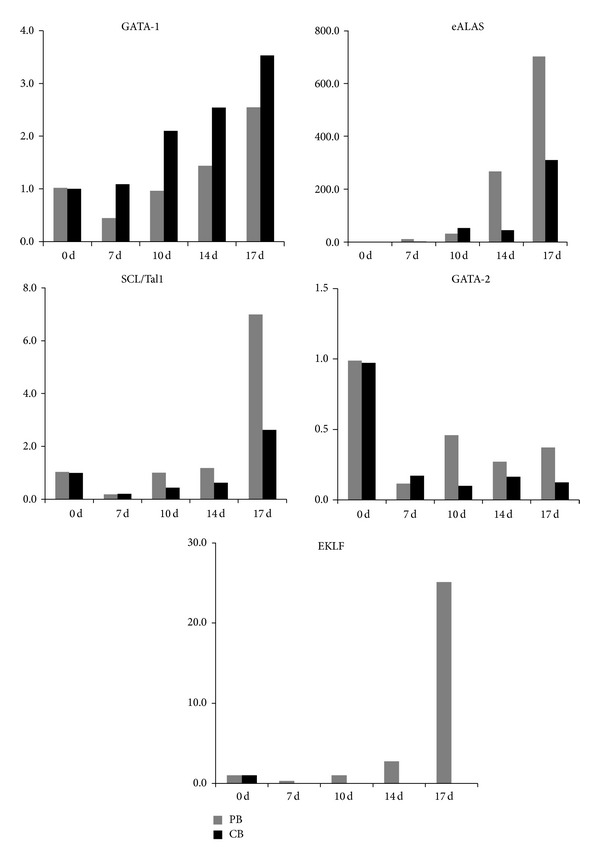
Erythrocyte-specific gene expression in mPB and CB cells. At the end of each phase of culture, cells were harvested, and total RNA was extracted for quantitative PCR. The expression of GATA-1, GATA-2, eALAS, EKLF, and SCL/Tal1 was measured by real-time PCR. The results show an increasing pattern for the GATA-1 transcript and a decreasing pattern for GATA-2, which did not significantly differ between mPB and CB cells. EKLF, eALAs, and SCL/Tall expression levels increased during differentiation but were significantly greater in mPB-derived erythrocytes than in those from CB.

From our data, we can see different patterns in the differentiation of mPB- and CB-derived CD34+ cells. Our data clearly show that GATA-1 expression gradually increases during erythrocyte differentiation, especially in CB cells, while GATA-2 expression gradually decreases following cell maturation. The erythrocyte-specific isoforms ALAS and SCL/Tall are upregulated during erythrocyte differentiation and, in particular, show higher levels in mPB-derived cells than in CB-derived cells. Only one factor, EKLF, which is a *β*-globin gene transcription factor, increased during erythrocyte differentiation in mPB cells but, in contrast, decreased during differentiation of CB-derived cells (Figure 4). These results clearly demonstrate that mPB-derived CD34+ cells differentiate faster into erythrocytes with Hb-*β* production than CB cells. At the same time, under these* in vitro* culture conditions, CB-derived CD34+ hematopoietic cells exhibit higher multipotency than mPB cells.

### 3.4. Different Hemoglobin Type Development in mPB- and CB-Derived CD34+ Cells

**Figure 5:**
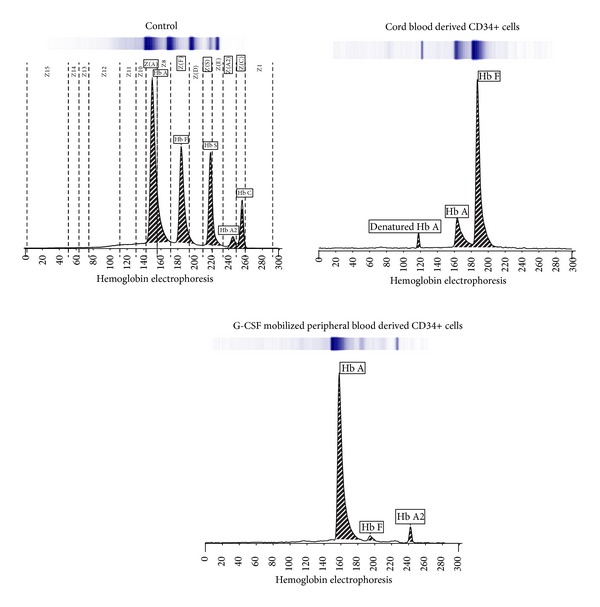
Electrophoretic determination of main hemoglobin subtypes in differentiated mPB and CB cells. Capillary zone electrophoresis shows distinct patterns of hemoglobin subtype expression between differentiated mPB and CB cells. About 80% of the hemoglobin produced by CB-CD34-derived erythrocytes is HbF, while only 17.5% is HbA. However, 95.5% of the hemoglobin produced by mPB-CD34-derived erythrocytes is HbA.

Over 80% of the hemoglobin produced by CB-derived CD34+ cells was hemoglobin subtype HbF, while only 17.5% was subtype HbA. Over 95% of the hemoglobin generated by mPB-derived cells was subtype HbA (Figure 5).

### 3.5. Similar Hemoglobin Dissociation Curve with Mature RBCs from mPB and CB Cells

**Figure 6:**
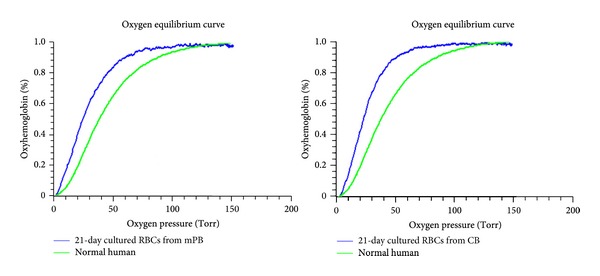
Hemox analysis in mPB and CB cells cultured for 21 days. Differentiated erythrocytes were harvested at the end of the 21 days of culture, and oxygen equilibria were measured by Hemox-Analyzer. Erythrocytes derived from mPB and from CB both show greater oxygenation abilities compared with normal human blood. There are no significant differences between differentiated mPB and CB cells.

While erythrocytes derived from each source have different combinations of HbA and HbF, similar hemoglobin dissociation curve was observed (Figure 6). Apart from this hemoglobin subtype variation, this result clearly shows that* in vitro* cultured RBCs can produce hemoglobin with oxygen binding and dissociation abilities equivalent to red blood cells produced* in vivo.*

## 4. Discussion

The shortage of blood supply and the ever-growing demand for blood transfusion represent a significant emerging issue in transfusion medicine. Shortage of donated blood and the risk of infection have created limitations in the availability of red blood cells available for transfusion. More recently, isolated CD34+ cells from human umbilical CB obtained from discarded maternity products and G-CSF-mPB obtained from healthy volunteers through leukapheresis have emerged as potential alternative sources of HSCs. The HSCs collected from both sources have high rates of proliferation and capacities for differentiation and create mature RBCs under the proper culture conditions through three phases of* in vitro* culture. We note that, though both types of stem cells show similar characteristics in general growth patterns with morphological and immunophenotypic changes, they show different characteristics in gene expression levels and hemoglobin subtype production.

Our results show that both mPB- and CB-derived CD34+ cells began to proliferate extremely quickly during 7–14 days of culture. However, both cell sources produce similar total cell numbers after 21 days of culture. And morphological changes in differentiated cells from both sources were similar, and no remarkable differences were found during the culture except when comparing cell differentiation rates. The mPB cells seemed to mature earlier than CB cells. These results show that mPB cells, from circulating blood, possess more progenitor cells than CB and have the ability of rapid differentiation into the mature RBCs. Following cell growth at the end of each cell culture phase, mPB- and CB-derived CD34+, GPA, and CD70 (transferrin receptor) expression showed similar patterns in flow cytometry analyses. These data are consistent with previous results regarding erythrocyte induction mechanisms [8, 14]. To confirm whether the initial exposition of cells is due to different sources derived differentiation, gene expression profiles were analyzed by quantitative RT-PCR. GATA-1 is a transcription factor that determines erythroid differentiation, survival, and *β*-globin gene expression [15]. GATA-2 inhibits GATA-1 function. GATA-2 expression exhibited a downregulation following erythropoietin stimulation, and its levels were higher in cultured mPB cells. This can be explained in earlier differentiation into mature RBCs in mPB source cells than in CB. This finding is also consistent with the result of differential counts that mPB showed faster shift from pronormoblasts to orthochromatic normoblasts in comparison to CB. Ikonomi et al. have previously shown that GATA-2 preferentially increases *γ*-globin gene expression, indicating that the prolonged expression of GATA-2 contributes to the early increase in *γ*-globin in CB [16]. EKLF binds specifically to the *β*-globin promoter and is critical in establishing chromatin structure for high-level *β*-globin transcription via its acetylation by CREB binding protein [17]. SCL/Tall is required for the progression of erythroid differentiation, and enforced expression of SCL/Tall increases *β*-globin expression and BFU-E and CFU-E production [18]. Because these transcription factors are closely related to increased *β*-globin gene expression, changes in their expression may account for the delay and reduction of *β*-globin expression in CB-derived differentiated cells. The HbA- and HbF-related gene expression tests exhibit different expression levels depending on the cell source. We cultured mPB- and CB-derived CD34+ cells through three phases, harvested them after 17 days of culture, and analyzed them by hemoglobin type testing. These results clearly demonstrated different subtypes of hemoglobin expressed by mPB- and CB-derived mature cells. CB-derived cells mostly express HbF and mPB-derived cells mainly express HbA, but, based on these data, the original sources of these cells appear to possess different propensities for hemoglobin production patterns. These results demonstrate that hemoglobin subtypes are not related to culture conditions and culture time but are strongly affected by the source material. To evaluate the function of the differentiated mPB and CB cells, CD34+ cells derived from each source were expanded and differentiated to large cell numbers of up to a total of 5 × 10^7^ cells, and oxygen equilibria were measured by Hemox-Analyzer. From the result, the oxygen dissociation curves indicate that the cells from both sources do not significantly differ from one another with respect to hemoglobin function. Although the types of hemoglobin expression differed between CB- and mPB-derived mature cells, the oxygen binding and dissociation curves may be similar due to variation among adult type hemoglobin, fetal type hemoglobin, or mixture of both types* in vitro* culture processing after 17 days of culture. The cultured cells, indeed, have slightly greater deoxygenation functionalities compared with normal cells, which showed the immaturity in shift to left.

In summary, mPB and CB are undoubtedly excellent sources for mature RBC production and may be key in contributing to a solution for the RBC supply shortage problem. Our study shows that, despite similar phenotypes and functionalities following erythrocyte maturation, the two are discrete in that they show different Hb types and gene expression levels. This study demonstrates distinctions that should be taken into account when choosing the source of HSCs for artificial mature RBC production form stem cells.

## Figures and Tables

**Table 1:** Real-time polymerase chain reaction primers.

Gene	Primer sequence
*β*-actin	
Forward primer Reverse primer	5′-ATTGGCAATGAGCGGTTC-3′ 5′-GGATGCCACAGGACTCCAT-3′

GATA-1	
Forward primer Reverse primer	5′-CACTGAGCTTGCCACATCC-3′ 5′-ATGGAGCCTCTGGGGATTA-3′

GATA-2	
Forward primer Reverse primer	5′-GGCAGAACCGACCACTCATC-3′ 5′-TCTGACAATTTGCACAACAGGTG-3′

eALAS	
Forward primer Reverse primer	5′-GATGTGAAGGCTTTCAAGACAGA-3′ 5′-GGAAAATGGCTTCCTTAGGC-3′

EKLF	
Forward primer Reverse primer	5′-ATCGAGTGAAGAGGAGACCTTCC-3′ 5′-TGAAGATACGCCGCACAACTT-3′

SCL/Tal1	
Forward primer Reverse primer	5′-ACACACAGGATGACTTCCTC-3′ 5′-CCCATGTCCTGCGC-3′
